# Altered and Agitated due to Hypertension: A Case of Posterior Reversible Encephalopathy Syndrome

**DOI:** 10.7759/cureus.8810

**Published:** 2020-06-24

**Authors:** Raza Kazmi, Emily Clark, Vir Singh, Michael Falgiani, Latha Ganti

**Affiliations:** 1 Emergency Medicine, Ocala Regional Medical Center, University of Central Florida College of Medicine, Ocala, USA; 2 Emergency Medicine, University of Central Florida College of Medicine, Orlando, USA; 3 Emergency Medicine, Ocala Regional Medical Center, Ocala, USA; 4 Emergency Medicine, Envision Physician Services, Nashville, USA; 5 Emergency Medicine, University of Central Florida College of Medicine/Hospital Corporation of America Graduate Medical Education Consortium of Greater Orlando, Orlando, USA; 6 Emergency Medical Services, Polk County Fire Rescue, Bartow, USA

**Keywords:** posterior reversible encephalopathy syndrome (pres)

## Abstract

The authors present a case of posterior reversible encephalopathy syndrome in a patient on nutritional supplements. The presentation and emergency management are discussed.

## Introduction

Posterior reversible encephalopathy syndrome (PRES), as the name suggests, is a constellation of symptoms caused by reversible ischemia most commonly of the posterior cerebral vasculature [1]. Various terminologies have been used to describe this condition, including "reversible posterior leukoencephalopathy syndrome" and "reversible posterior cerebral edema syndrome" among others [2].

## Case presentation

A 55-year-old male presented to the emergency department (ED) via emergency medical services (EMS) with a chief complaint of altered mental status, seizure-like activity, and agitation.

The history of presenting illness and other information was obtained from EMS and the patient’s wife, as the patient was agitated on arrival to the ED. According to the patient’s wife, prior to arrival the patient had been complaining of the "worst headache of his life" after sexual intercourse, associated with vision changes and neck pain. He then suddenly began to have seizure-like activity, which his wife described as full body spasms. Immediately after this episode, the patient became altered. EMS stated that when they arrived at the home the patient was found to be sitting in a chair, appeared to be postictal, and then quickly became combative.

The patient had no significant past medical history, including no prior seizures. He endorsed similar headaches after intercourse over the last 10 years. The patient occasionally smoked marijuana and had a longstanding history of taking two over-the-counter nutritional supplements, according to the label recommendations (Table 1).

**Table 1:** The patient's nutritional supplements details

Name of supplement	Ingredients	Serving size
Ultra Fast Keto Boost	62 mg calcium beta hydroxybutyrate, 32 mg magnesium beta hydroxybutyrate, 10 mg sodium beta hydroxybutyrate. Above as components of an 800 mg “Go BHB© Proprietary Blend”. Other ingredients included gelatin, rice flour, stearate, and silicon dioxide.	Two capsules daily
Clear Nails Plus Extra Strength Nail Formula	200 mg/6 billion CFU “probiotic fungus blend”: Lactobacillus rhamnosus, Lactobacillus plantarum, Bifidobacterium longum, Lactobacillus acidophilus, Lactobacillus salivarius, Bifidobacterium lactis, Bifidobacterium bifidum, Lactobacillus fermentum, Lactobacillus reuteri, Inulin. 450 mg of turmeric and 2.5 mg “BioPerine™, a proprietary piperine derivative Other ingredients include gelatin, maltodextrin, medium chain triglycerides, magnesium stearate, and rice flour.	One capsule daily

A complete review of systems was unobtainable secondary to the patient’s condition. Vital signs obtained included a pulse of 138 beats per minute, a blood pressure of 200/125 mmHg, and a blood glucose of 138 mg/dL. He remained combative, requiring medics to hold down each of his extremities to allow for emergent evaluation. Other than tachycardia and psychomotor agitation, the patient’s physical exam was grossly normal. A comprehensive neurologic assessment was unable to be performed prior to sedation due to agitation. The patient was subsequently intubated for the safety of the patient and staff, and to allow for a complete emergency diagnostic workup to be performed. Rocuronium and etomidate were utilized for intubation, and the post-intubation sedation was achieved with propofol.

Differential diagnosis included meningitis, encephalitis, aspiration pneumonia, sepsis, subarachnoid hemorrhage, subdural hemorrhage, undiagnosed seizure disorder, and drug intoxication. The initial diagnostic workup included a complete blood count (CBC), a comprehensive metabolic panel (CMP), a lactic acid level, an arterial blood gas (ABG), a urine drug screen, ethanol, acetaminophen, and salicylate levels. Imaging included CT of the brain and CT angiography (CTA) of the brain, neck, and chest.

Significant laboratory findings included an arterial pH of 7.27, HCO_3 _of 18.9 mEq/L with a base excess of -8 at an FiO_2_ 60%, lactic acid of 5.95 mmol/L, basic metabolic profile significant for carbon dioxide level (CO_2_) of 11 mmol/L, white blood cell (WBC) count of 18.9 x 10^3^/mm^3^ with neutrophil count of 12.3%. Toxicological screen was positive for tetrahydrocannibinol. Non-contrast CT of the brain, CTA of the head and neck, and CT brain perfusion were all without abnormal finding. CTA of the chest was significant for bibasilar pneumonia, which may have reflected aspiration.

**Figure 1:**
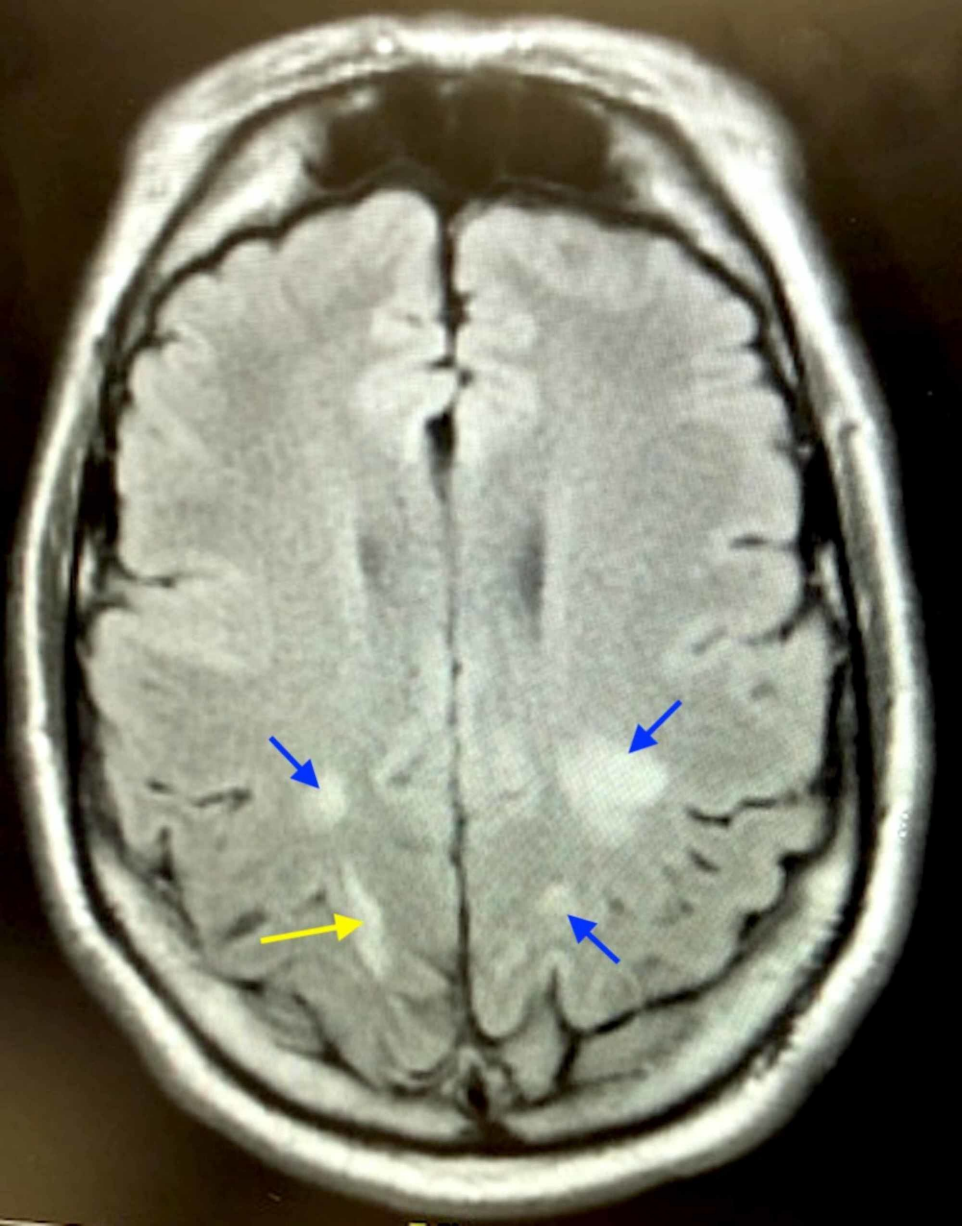
MRI demonstrating bilateral parietal and occipital subcortical T2 hyperintensities (blue arrows) with right occipital artifactual restricted diffusion (yellow arrow). No evidence of acute infarct, hemorrhage, hydrocephalus, or mass.

The patient was subsequently admitted to the intensive care unit (ICU). In the ICU, the patient was hypotensive upon arrival. Sedation was discontinued, a central venous catheter was placed, and vasopressor support was initiated with norepinephrine. An electroencephalogram (EEG) was performed and did not show any epileptic spike and wave morphology. MRI of the brain was subsequently obtained, which showed parietal and occipital changes consistent with PRES (Figure 1). These radiologic findings and diagnosis could be clinically correlated with the patient’s poorly controlled hypertension.

His hypertensive state in the ICU was managed originally with intravenous (IV) labetalol boluses, and subsequently with a nicardipine infusion. Piperacillin/tazobactam and azithromycin were administered for pneumonia, which were later switched to vancomycin and cefepime. The patient was extubated on inpatient day 2 and downgraded to the neurology floor on day 3. Neurology established a target blood pressure goal of systolic blood pressure below 140 mmHg. The patient was advised about the importance of blood pressure monitoring at home and connected with a primary care physician with whom to follow up within one week of discharge. He was discharged home on daily lisinopril.

## Discussion

PRES is a neurological syndrome that usually evolves over hours, and typically presents with seizures, disturbed vision, headache, altered mental status, and encephalopathy accompanied by markedly elevated blood pressure [3]. PRES often develops in patients who are on cytotoxic medications, or in the context of renal disease, sepsis, or autoimmune disorders.

There are currently two theories as to the cause of PRES. One theory is that rapidly increasing blood pressure in hypertensive urgency or emergency exceeds the upper autoregulatory limits of cerebral perfusion leading to vasogenic edema and leakage from cerebral vasculature. However, not all patients with PRES have elevated blood pressures at symptom onset, and approximately 30% of patients have normal or only slightly elevated pressures [2]. The second etiologic theory for PRES is that it is triggered by endothelial dysfunction due to circulating toxins. This theory is derived from the observation that PRES often occurs in patients who are undergoing treatment with cytotoxic medications, and have sepsis or renal disease. Circulating toxins in these disease states could alter the integrity of the vasculature of the cerebral circulation allowing for vasogenic edema and vascular leakage. 

Suggested diagnostic criteria include acute onset of neurological symptoms, focal posterior vasogenic edema on imaging, and reversibility of clinical and/or imaging findings [4]. MRI classically shows evidence of symmetrical vasogenic edema in the posterior regions, often the parieto-occipital area. The common MRI features seen in PRES are summarized in Table 2 [5,6].

**Table 2:** MRI findings in PRES ADC, apparent diffusion coefficient; DWI, diffusion-weighted imaging; FLAIR, fluid-attenuated inversion recovery; PRES, posterior reversible encephalopathy syndrome

MRI findings in PRES
Parieto-occipital dominance
Most often bilateral, and symmetrical
Vasogenic cerebral edema
Holohemispherical watershed distribution
Superior frontal sulcus
Subcortical white matter
Hyperintense T2-weighted and FLAIR sequences
DWI usually normal
Increased ADC values reflective of vasogenic cerebral edema

Treatment of PRES is directed by symptoms and the underlying condition. Hypertension guidelines follow those of hypertensive emergency, and often seizure prophylaxis is warranted. When possible, treatment is aimed at removing underlying pathology or inciting factor. The patient outcomes are based on their underlying condition as PRES symptoms, and lesions seen on imaging are often reversible.

In our patient case, we found acute onset encephalopathy, which is a common presentation of PRES. He also had profound hypertension with the worst headache he had ever experienced which also follows with PRES syndrome. Our case is also the first documented case of PRES associated with the use of marijuana, keto, and antifungal supplements. Although use of these substances may be unrelated, it is important to document such associations in the event that a correlation may be established in the future. Thus, in our case, profound hypertension likely caused vasogenic edema and vascular leakage leading to PRES, which was further supported by posterior edema seen on MRI obtained during the hospital course.

## Conclusions

It is imperative for emergency physicians to know about the clinical symptoms and radiological findings, diagnosis, and treatment of patients with PRES to initiate symptomatic and preventative treatment early in the disease process to possibly reduce complications and abbreviate syndrome duration.
